# Near-Horizon Carnot Engines Beyond Schwarzschild: Exploring Black Brane Thermodynamics

**DOI:** 10.3390/e27050491

**Published:** 2025-05-01

**Authors:** Lotte Mertens, Jasper van Wezel

**Affiliations:** Institute for Theoretical Physics Amsterdam, University of Amsterdam, Science Park 904, 1098 XH Amsterdam, The Netherlands; vanwezel@uva.nl

**Keywords:** thermodynamics, Carnot engine, black hole thermodynamics, black brane

## Abstract

Sadi Carnot’s seminal work laid the foundation for exploring the effects of thermodynamics across diverse domains of physics, stretching from quantum to cosmological scales. Here, we build on the principles of the original Carnot heat engine, and apply it in the context of a particular toy model black brane. This theoretical construct of an effectively two-dimensional, stable, and stationary gravitational object in four-dimensional spacetime derives from a hypothetical flat planet collapsed under the influence of gravity. By constructing a thermodynamic cycle involving three such black branes, we explore the possibility of energy extraction or mining, driven by the temperature gradients and gravitational potential differences characteristic of curved spacetime. Analytic solutions obtainable within this toy model illuminate key aspects of black hole thermodynamics in general, particularly for spacetimes that are not asymptotically flat. Central to these findings is the relation between gravitationally induced temperature ratios and entropy changes, which collectively offer a novel perspective on obtainable energy transfer processes around gravitational structures. This analysis highlights potential implications for understanding energy dynamics in gravitational systems in general, including for black hole evaporation and experimentally implemented black hole analogues. The presented findings not only emphasise the universality of the thermodynamic principles first uncovered by Carnot, but also suggest future research directions in gravitational thermodynamics.

## 1. Introduction

Sadi Carnot’s seminal work “Réflexions sur la puissance motrice du feu”, laid the foundation for applications of thermodynamics across all domains of physics, stretching from microscopic quantum scales all the way to cosmological processes [1]. Here, we focus on a version of Carnot’s heat engine arguments applied to modern black hole thermodynamics. The idea of mining black hole radiation by transferring energy between two infinitely separated Schwarzschild black holes in a thermodynamic or Carnot-engine-type cycle has been well-established [2,3]. This seminal gedanken experiment combining classical thermodynamics and black hole physics revealed a direct connection between energy-transfer processes and spacetime geometry, and gained prominence following the foundational work of Hawking and Bekenstein, who demonstrated that black holes possess thermodynamic properties, including entropy and temperature [4,5,6]. Together, these works embed classical thermodynamics into the framework of general relativity and quantum field theory, underscoring the universality of Carnot’s legacy.

Building on this rich tradition, here we propose a similar thermodynamic cycle in a particular, effectively two-dimensional black hole system, consisting of a theoretical toy model for a black brane. Unlike traditional Schwarzschild or Kerr black hole geometries, this black brane has the distinct advantage that it allows all of the involved calculations to be done analytically, in closed form. Consequently, the toy model serves as a theoretical lens for probing the interplay between entropy, temperature, and energy within curved spacetime geometries. It applies in particular to spacetimes that are not asymptotically flat, but general conclusions will be highlighted.

To obtain a closed Carnot cycle, it turns out we need to employ a system containing three black branes, as opposed to the classic setup containing two Schwarzschild black holes [2]. Focusing on this richer setup offers several advantages. Notably, it is exactly solvable but non-trivial, a rare combination in the study of black hole thermodynamics. Moreover, the interplay of temperature gradients connecting the multiple black branes in this configuration serves as a natural platform to explore fundamental questions about energy extraction, radiation transfer, and the limits of thermodynamics in curved spacetime. The importance of temperature, both as a physical observable and a theoretical construct, remains a hot topic throughout general relativity, underlying, for example, black hole evaporation and the holographic principle. By extending thermodynamic cycles to a system of black branes, these key ideas can be explored in a particularly accessible setting, allowing a fresh view on gravitational thermodynamics.

In this article, we will first provide a detailed overview of the toy model black brane, highlighting the relevance of its properties to thermodynamic analyses. We then analyse a Carnot engine-based thermodynamic cycle in a setup containing three black branes. In this context, we highlight the implications of energy transfer and efficiency within the Carnot cycle in curved spacetime. We conclude by discussing the significance of the obtained results within the broader context of black hole thermodynamics and general relativity.

## 2. The Toy Model Black Brane

The proposed Carnot cycle will be defined in a system comprising three entities that we term ‘toy model black branes’. They are effectively two-dimensional analogues of the usual point-like black hole singularities in four-dimensional spacetime. To ensure that the toy model black brane contains a massive, sheet-like singularity, we explicitly construct it from the gravitational collapse of a toy model, non-singular planetary body extending infinitely in two spatial dimensions (denoted *y* and *z*), while maintaining a finite width along the third (written *u*). Such a board-like, infinitely large object can be described by a non-singular, static solution to Einstein’s Equations [7]. The metric for such a body, expressed in its proper frame, is given by:

(1) $$\begin{array}{cc} g_{vv}=-1/g_{uu} & =-\gamma u\tanh\left(\frac{u}{a}\right)\\ g_{yy}=g_{zz} & =\tanh{\left(\frac{\left|u\right|}{a}\right)}^{-1}. \end{array}$$

Here, *v* represents the temporal coordinate, and *a* is a measure for the finite width of the planetary object. The centre of mass is located at u=0, and the metric is symmetric under reflection in the u=0 plane. All details regarding the associated Christoffel symbols, the Riemann tensor, the Ricci tensor, the Ricci scalar, and the pressure and density of this static solution to Einstein’s equations can be found in Appendix A.

When subjected to perturbations, the static planetary configuration becomes dynamically unstable. As long as the perturbation maintains the invariance of the metric in the *y* and *z* directions, the gravitational pull in the *u*-direction induces a uniform collapse, eventually compressing the planetary width *a* to infinitesimal values (assuming interactions dissipate the involved kinetic energy). The resulting state is characterised by a singularity at u=0, where the mass density diverges. This singularity, uniform along the *y* and *z* directions, defines the toy model black brane. The metric for this collapsed configuration corresponds to the well-known Rindler metric everywhere except within the infinitesimal region $u\in \lim_{a\to 0}\left[-a,a\right]$, where the singularity resides. Outside this region, the metric has non-zero components [8]:

(2) $$\begin{array}{ccc} \; & g_{vv}=-1/g_{uu}=-\gamma \left|u\right|\; & g_{yy}=g_{zz}=1. \end{array}$$

In this configuration, a horizon emerges at u→0, thus rendering the sheet-like singularity naked. Its infinite spatial extent, however, causes spacetime to be divided into disconnected regions despite the singularity being naked. Furthermore, as explicitly derived in Appendix A, the black brane features a finite and non-zero surface gravity $\kappa =\gamma /2=2\pi G\rho_{0}$, where we defined $\rho_{0}$ to be the mass density of the singular sheet [9]. Translational symmetry guarantees the surface gravity and mass density to be constant along the horizon.

Unlike conventional Schwarzschild black holes, the toy model black brane permits a global definition of quantum fields across all positions with u≠0. The resulting mathematical tractability of field-theoretic calculations enables the derivation of analytic solutions for numerous phenomena, providing a general framework for exploring thermodynamic processes in curved spacetime. Moreover, the toy model black brane allows for the construction of potential experimental analogues, offering a platform for empirical validation of any predictions [10,11,12,13].

## 3. A Black Brane Heat Pump

**Figure 1:**
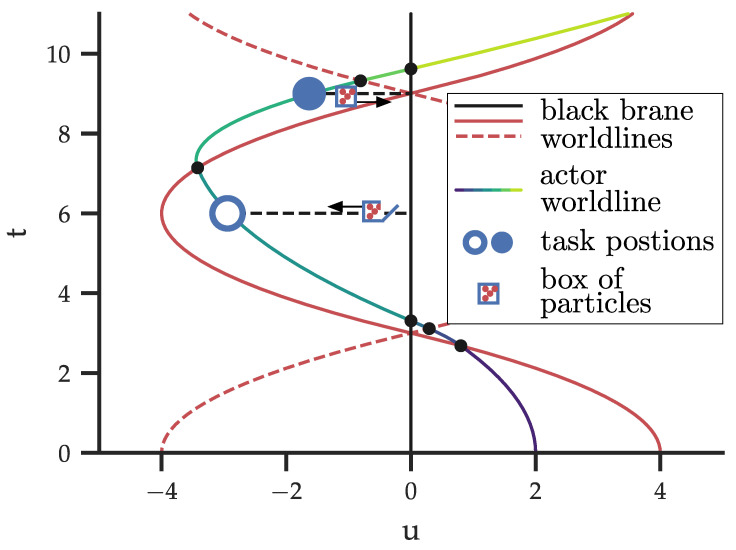
**Carnot engine-based thermodynamic cycle for toy model black branes.** Schematic setup of the time evolution encountered in the tripartite black system using coordinates *u* (distance measured by an observer static with respect to the central black brane) and *t* (time measured by the free-falling observer). The solid and dotted red lines shows the two outer branes with equal mass density $\rho_{2}$, and maximal position |u|=4. The solid black line represents the central black brane with mass density $\rho_{1}$. The multi-coloured line indicates a free-falling observer, starting from an initial position at u=2. The blue open ($u=d_{○}$) and closed ($u=d_{●}$) circles are the points at which the free-falling observer performs a task. The black dots indicate instances at which the observer passes through a brane.

To explore the thermodynamics of black branes, we construct a Carnot cycle involving three distinct black branes, as depicted schematically in Figure 1.

**Actors in the thermodynamic cycle**—At the initial time v=0, the system consists of a central black brane situated at u=0 (indicated in Figure 1 by a black worldline), and two outer black branes (shown by red solid and dashed worldlines) positioned symmetrically with respect to the central brane, at u=±b. The central brane is characterised by mass density $\rho_{1}$, while the outer branes both have mass density $\rho_{2}$. Due to the symmetry of the configuration, the gravitational fields of the outer branes cancel within the region between them, leaving the gravitational potential of the central brane to dominate there.

The two outer branes oscillate around the central brane under its gravitational influence, periodically converging at u=0. For the sake of simplicity, we assume that the particles comprising the different branes are entirely non-interacting and can freely traverse the black brane singularity at u=0. While non-physical, this assumption does not affect the thermodynamic analysis, while enabling a more intuitive visualization of the cycle. A freely falling observer, acting as an agent, interacts with the system by performing tasks that either consume or generate work. This interaction is facilitated using a massless box of particles, which the agent lowers or raises at specific instances within the temporally varying gravitational potential. In Figure 1, the observer’s worldline is colour-coded to represent the metric changes perceived by the observer. The tasks performed at events indicated by blue circles constitute a Carnot engine similar to that proposed for the mining of spherical black holes [2].

In the classic formulation of black hole mining protocols, extracting particles precisely from the horizon would result in a perpetual motion machine [3]. However, this is unachievable in practice, since adiabatically lowering the box to the horizon requires infinite energy and time. To circumvent this complication, we can consider a second observer, following a reverse cycle compared to the first one. If particles are exchanged between the reservoirs carried by the two observers, a closed thermodynamic cycle can be maintained throughout and traversed in finite time. As shown below, this still allows for a well-defined temperature ratio between horizons associated with different black branes to be established, in direct analogy with the original (perpetual motion) mining protocol.

**Figure 2:**
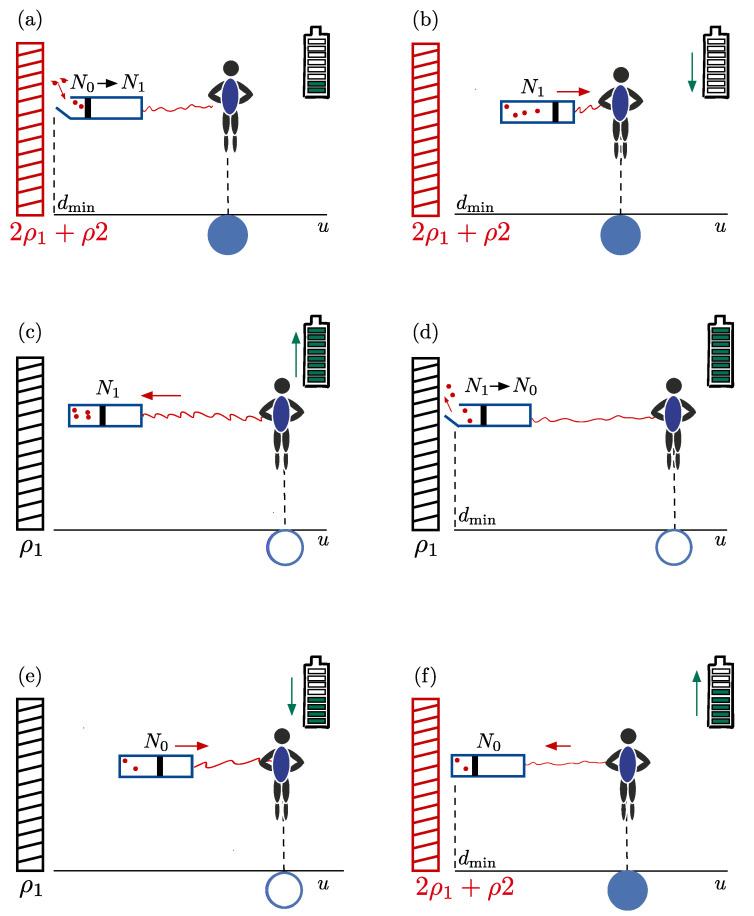
**Cartoon depiction of the tasks performed during a single thermodynamic cycle.** The letters for each subfigure indicate the corresponding stages of the Carnot cycle discussed in the text. In each stage, the observer is shown alongside the box of particles they raise and lower towards the brane, the battery they use to do work or store energy with, the position they are located at, and the instantaneous mass density of the black brane they encounter. The arrows denote the direction of lowering or raising the box.

**Stages of the thermodynamic cycle**—The thermodynamic cycle consists of six distinct steps, each characterised by a task to be performed by the free-falling observer. A cartoon relating the different steps is provided in Figure 2, and a list of the symbols employed in the description can be found in Table 1. The stages comprising one full cycle consist of the following:

*The initial position*—The actor begins at the closed blue dot in Figure 1 and Figure 2, positioned at *d*_●_. At this particular instance of time, the outer branes coincide with the central brane at u=0, effectively forming a single brane with high mass-density. The observer carries an energy storage device (a battery), which is partially charged (e.g., it is shown to be 1/4 full in the cartoon in Figure 2). The observer also holds a closed (massless) box at a distance $d_{\min}$ from the central brane’s horizon, containing $N_{0}$ particles with total energy $U_{0}$.

*Process (a): Heat Absorption*—At the initial position, the observer remotely opens the box, allowing additional particles to flow in. These particles originate either from the black brane horizon (in the perpetual motion formulation) or from the reservoir of a second observer (in the closed-system formulation). Heat $Q_{H}$ is transferred into the box, increasing the system’s energy to $U=U_{0}+Q_{H}$ without performing work.

*Process (b): Adiabatic Raising*—The observer then adiabatically raises the box to its own position *d*_●_. Adiabatic isolation ensures there is no heat exchange (ΔS=0 and Q=0). However, pulling against the gravitational potential requires work $W_{\mathrm{in},1}$, draining the observer’s battery. The system’s energy is $\Delta U=-W_{\mathrm{in},1}$.

*Free-fall*—In the subsequent stage, the observer free-falls toward the central brane along with their box, traversing an oscillatory trajectory dictated by the gravitational dynamics of the system. Meanwhile, the outer branes separate due to their own oscillatory motion. At some point during their free fall, the observer moves through an outgoing mass sheet, but continues to free-fall unabated. Upon arriving at position $d_{○}$, the observer will perform the next set of tasks. During the entire free fall, the internal energy and particle number in the box remain unchanged.

*Process (c): Adiabatic Lowering*—At this point, the box will be adiabatically lowered to $d_{\min}$. Gravitational work $W_{\mathrm{out},1}$ increases the battery’s charge during this process. The energy change within the box is $\Delta U=W_{\mathrm{out},1}$, with constant entropy and no heat transfer.

*Process (d): Heat Release*—Next, particles are allowed to flow out of the box until the initial number of particles, $N_{0}$, is restored. This reduces the entropy contained in the box, and releases heat $Q_{C}$. The system’s energy changes by $\Delta U=-Q_{C}$.

*Process (e): Adiabatic Raising*—The box is then pulled up to *d*_●_, performing work $W_{\mathrm{in},2}$ against the gravitational potential and partially draining the battery. No heat exchange occurs (Q=0 and ΔS=0), thus $\Delta U=W_{\mathrm{in},2}$.

*Free-fall*—After the box is pulled up, the box and observer free-fall again, and pass through a mass sheet before they return to position *d*_●_. The internal energy does not change during this process.

*Process (f): Adiabatic Lowering*—Finally, lowering the box to $d_{\min}$ extracts work $W_{\mathrm{out},2}$, and completes the cycle. The battery ends up with a net charge increase, indicating that work has been gained during the cycle. The energy of the box, meanwhile, returns to its initial value $U_{0}$, satisfying $U_{\mathrm{final}}-U_{0}=Q_{H}-W_{\mathrm{in},1}+W_{\mathrm{out},1}-Q_{C}+W_{\mathrm{in},2}-W_{\mathrm{out},2}=0$.

This sequence can be repeated cyclically, with the observer systematically manipulating the box to extract work. The extracted work is a direct consequence of the gravitational potential differences encountered in the various black brane configurations, and we will discuss the implications of its existence in the next section.

**Figure 3:**
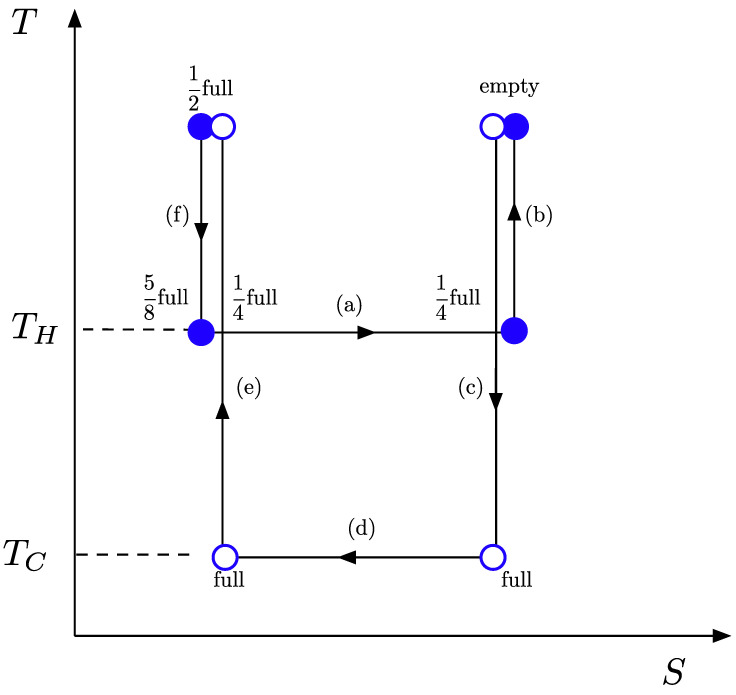
**Entropy–temperature diagram of the Carnot cycle toy model black branes.** The various stages of the cycle are labelled by letters corresponding to the processes described in the main text. Notice that free-fall processes conserve both temperature and entropy and, therefore, do not correspond to line segments in the S−T diagram. Possible values for the instantaneous charge of the battery carried by the observer are indicated at various point encountered during a full cycle, and correspond to values indicated in the cartoon of Figure 2. The isothermal processes (heat absorption and release) correspond to horizontal segments, while adiabatic processes (raising and lowering the box) correspond to vertical segments.

**The Carnot cycle**—The closed thermodynamic cycle described above can be depicted on an entropy-temperature (S−T) diagram, as shown in Figure 3. The labelled arrows indicate the processes encountered during a full cycle, and possible values for the instantaneous charge of the battery at the end of each process (corresponding to those shown in the cartoon depiction of Figure 2) are indicated.

The thermodynamic temperatures indicated in Figure 3 are defined by T=Q/ΔS, applied to the isothermal processes (a) and (d). The changes in entropy associated with the heat transfers $Q_{H}$ and $Q_{C}$, encountered close to the black-branes with larger and smaller mass density, respectively, are denoted $\Delta S_{H}$ and $\Delta S_{C}$. These changes in entropy are determined entirely by the change in the number of particles in the box, and they are, therefore, equal. The temperature ratio $T_{H}/T_{C}$ then becomes:

(3) $$\begin{array}{c} \frac{T_{H}}{T_{C}}=\frac{Q_{H}/\Delta S_{H}}{Q_{C}/\Delta S_{C}}=\frac{Q_{H}}{Q_{C}}. \end{array}$$

The presence of this finite ratio defines the thermodynamic process to be a Carnot engine, a cyclic process from which work is extracted. From the definition of temperature as given by Claussius [14], we then know that the heat consumed during each stage of the cycle is proportional to the work gained. In turn, the work that is performed on the actor (or the work the actor performs on the box) is due to the lowering and raising of the box in the gravitational potential. As this is done adiabatically, the work must equal the change in Killing energy $E=N\sqrt{g_{tt}\left(d\right)}E_{0}$, with rest energy $E_{0}$:

(4) $$\begin{array}{c} \frac{T_{H}}{T_{C}}=\frac{\left(N_{1}-N_{0}\right)E_{0}\sqrt{\gamma_{H}\left(d_{●}-d_{\min}\right)}}{\left(N_{1}-N_{0}\right)E_{0}\sqrt{\gamma_{C}\left(d_{○}-d_{\min}\right)}} \end{array}$$

Here, $\gamma_{C}=4\pi G\rho_{1}$ and $\gamma_{H}=4\pi G\left(2\rho_{2}+\rho_{1}\right)$ are defined by the mass densities of the single or combined black brane systems. At $d_{●}=d_{○}$, we can define a position independent temperature ratio of

(5) $$\begin{array}{c} \frac{T_{H}}{T_{C}}=\sqrt{\frac{\gamma_{H}}{\gamma_{C}}}. \end{array}$$

for the temperatures of the boxes during the isothermal processes. Importantly, this ratio is independent of $d_{\min}$ and remains valid in the limit $d_{\min}\to 0$. This is the limit required for the original perpetuum mobile formulation of the mining procedure. In that context, it is often suggested to describe a temperature ratio associated with the horizons for two black holes of different mass. In the present black brane setup, we can likewise recognise the ratio of Equation (5) to be consistent with that of the Unruh temperatures $T_{H,C}=\alpha_{H,C}/\left(2\pi \right)=\sqrt{\gamma_{H,C}}/\left(4\pi \sqrt{\left|u\right|}\right)$ experienced by observers in the metric of Equation (2) undergoing local acceleration α in order to remain static with respect to the black brane singularity.

**On thermal equilibrium**—The semi-classical description of a heat engine based on black hole mining introduces a well-known interpretational conundrum related to entropy loss [15]. When particles are gradually lowered towards a horizon, their Killing energy approaches zero. Upon crossing the horizon, these particles then contribute to neither the black hole entropy, nor its mass. However, since the particles effectively vanish from the universe, the system appears to lose entropy overall, ostensibly violating the second law of thermodynamics.

The paradox is clarified by two crucial observations. First, the Killing energy reaches zero only at the horizon itself, and it is impossible for any static observer to lower a box of particles to the precise location of the horizon within finite time or energy. As a result, a thermodynamic cycle involving the addition of zero Killing energy particles to the black hole cannot be physically realised, thereby mitigating concerns of entropy loss. Second, even if such a cycle were possible, classical black holes—being perfect absorbers [15]—cannot achieve thermodynamic equilibrium with their surroundings. This renders the concept of temperature for classical black holes undefined and undermines the meaningful assignment of thermodynamic properties in such contexts.

The scenario changes if black holes radiate. Hawking radiation potentially enables black holes to emit energy and establish thermal equilibrium with their environment. In this case, the radiation flux could lend a hypothetical physical basis to the thermodynamic temperature used in black hole mining protocols.

By reframing the discussion to avoid reliance on zero Killing energy particles, an alternative interpretation emerges. Instead of depositing particles into the black brane, one can consider a system where particles are exchanged between two thermodynamic cycles operating in opposite directions. In such a configuration, the Carnot engine’s work originates solely from gravitational potential differences traversed during the cycle. This perspective maintains a classical interpretation: the ratio of temperatures encountered in the Carnot cycle directly reflects the gravitational potential differences or mass ratios of the involved black holes. Thus, the Carnot cycle functions as a precise measurement tool for the properties of the black hole system, rather than as a source of energy extraction.

In the present setup of a thermodynamic cycle based on black branes, the interpretation of the ratio of temperatures reflecting only a ratio of gravitational potentials gains additional credence. The Hawking temperature of the black brane vanishes identically [7], foregoing any possible interpretation in terms of thermodynamic equilibrium or radiation flux.

## 4. Conclusions

The toy model black brane, with its precise invariance and infinite extent in two spatial directions, does not constitute a realistic description of any actual gravitational object. It instead serves the purpose of making possible a thermodynamic analysis in a mathematically accessible setting constructed out of well-understood gravitational components, consistent with all known laws of nature.

Here, we connected the foundational ideas of Carnot thermodynamics with black brane systems, demonstrating the theoretical feasibility of a thermodynamic cycle involving three symmetrically arranged black branes. We explicitly showed how temperature gradients and gravitational potential differences can drive energy transfer during the thermodynamic cycle. The exact solvability of the black brane system provides theoretical clarity in an area often marred by approximations. It also shows that the idea of gravitational thermodynamic cycles can be applied to spacetimes that are not asymptotically flat, and yield the same type of temperature ratios as in their better-known asymptotically flat cousins.

Black hole thermodynamics is notoriously hard to probe or observe in astronomical settings. Recent progress has been made in simulating and experimentally implementing black hole analogues in a variety of platforms, including electronic systems, gravity waves in fluids, and optical setups [10,11,12,13]. Such gravity analogues are now close to allowing experimental implementation of heat engines and other black hole physics. Because of the invariance of the black brane described here under translations in two spatial directions, its implementation may be especially suitable for setups that naturally favour one-dimensional flows, such as electrons in quantum wires or waves propagating in a flowing fluids [10,13].

The principles encountered in the exact black brane Carnot cycle analysed here, offer insights into energy extraction processes in other gravitational systems, as well as experimental black hole analogues, and the fundamental nature of entropy and temperature in the context of general relativity. As we celebrate 200 years since Carnot’s groundbreaking insights, we reaffirm the timeless relevance of their thermodynamic principles, and their capacity to illuminate physics across all scales.

## Figures and Tables

**Table 1:** Symbols used to describe the thermodynamic cycle.

u=0	Location of central brane
$N_{0}$	Initial number of particles in box
$U_{0}$	Initial energy of particles in box
*d*_●_	Initial distance of actor to the central brane
	(closed blue dot in Figure 1 and Figure 2)
$d_{○}$	Actor to central brane distance in second position
	(open blue dot in Figure 1 and Figure 2)
$d_{\min}$	Minimal distance between box and central brane
$Q_{H/C}$	Heat transferred into/out of box
$W_{\mathrm{in}/\mathrm{out}}$	Work required to raise/lower box

**Figure A1:**
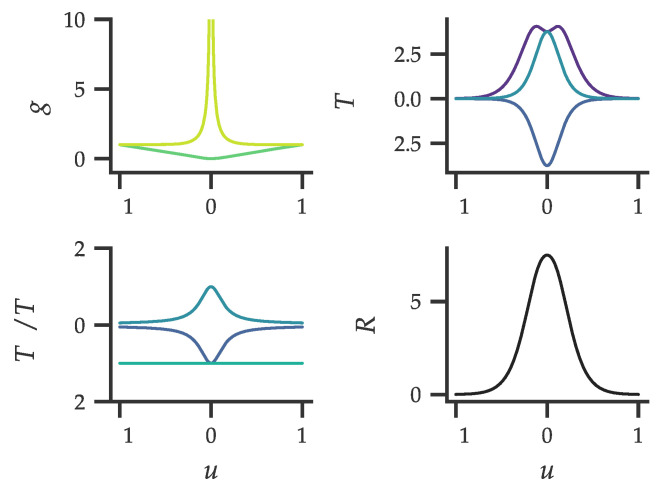
**Properties of the toy model planetary object.** The metric components for the board-like planetary object with γ=1 and a=0.2 are displayed in the **upper left** panel with the light green line showing $-g_{\nu \nu }$ and the dark green line $g_{uu}$. In the **upper right** panel, the density (ρ, top, purple line) and pressure ($P_{x}$ dark blue, $P_{y}=P_{z}$ light blue) are indicated, while the **lower left** panel shows the ratios between them with dark blue corresponding to $P_{x}/\rho$, light blue $P_{y}/\rho$, and the bottom turquoise line to $P_{x}/P_{y}$. The **lower right** panel displays the Ricci scalar.

**Figure A2:**
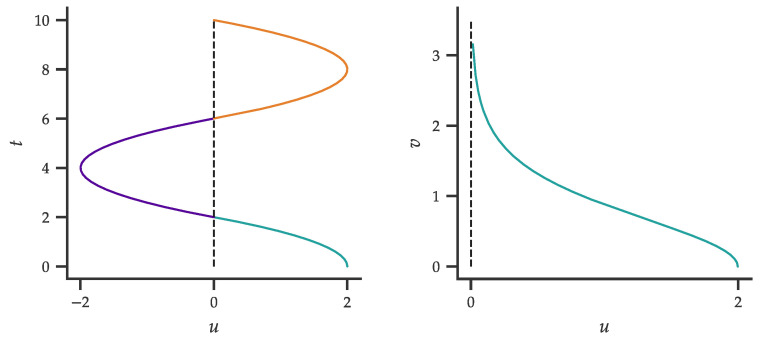
**Trajectories of massive in-falling test particles.** The geodesic paths for massive test particles are shown in different coordinate systems. We take γ=2 and consider the particle starting at rest at u=2. The **right** panel shows the trajectory expressed in the proper coordinates *u*, *v* of an observer at rest with respect to the singularity at u=0. The **left** panel shows the same trajectory, but with the temporal coordinate transformed to the proper time of the free-falling object itself. It can then be seen to reach the singularity in finite proper time. Trajectories in different colours are for different free-falling objects with initial conditions chosen to connect to one another at the singularity.

## Data Availability

The article includes all necessary data and information to recreate the results.

## Symbols

The following symbols are used in the manuscript (listed in order of appearance):

$g_{\mu ,\nu }$	the spacetime metric
*v*	a time-like coordinate
*u*, *y*, and *z*	space-like coordinates
*a*	width of the board-like, non-singular planetary object
γ	parameter determining the surface gravity of the planetary object
κ	the non-zero surface gravity of the sheet-like singularity
*G*	Newton’s constant
$\rho_{0}$	mass density of the sheet-like singularity
*b*	initial position of the outer singular sheets in the Carnot cycle
$\rho_{1}$ and $\rho_{2}$	mass densities of the inner and outer singular sheets in the Carnot cycle
*d*_●_ and $d_{○}$	positions at which the observer performs tasks
$d_{\min}$	minimal distance between the box and the central singular sheet
*N* and *U*	number and energy of particles in the box
*H*, *C*	indices indicating processes (a) and (d) of the Carnot cycle
1, 2	indices indicating processes (b) and (e) of the Carnot cycle
0 and final	indices indicating initial and final configurations
ΔS	change in entropy
*Q*	heat transfer
$W_{\mathrm{in},\;\mathrm{out}}$	work required to raise (lower) the box
ΔU	change in energy
*S* and *T*	entropy and temperature
*E*	Killing energy
α	acceleration

## Appendix A. The Infinite Board-like Planetary Object

This appendix details the static solution to the Einstein equations defining a toy model, board-like, non-singular planetary object extending uniformly towards infinity in two spatial dimensions. The toy model black brane discussed in the main text is the end-product resulting from the gravitational collapse of the planetary object described here. We start by defining its metric in the static coordinates of the planet itself:

(A1) $$\begin{array}{c} ds^{2}=-\gamma u\tanh\left(u/a\right)dv^{2}+1/\left(\gamma u\tanh\left(u/a\right)\right)du^{2}+\tanh{\left(|u|/a\right)}^{-1}\left(dy^{2}+dz^{2}\right). \end{array}$$

The planet has an infinite extent in the *y* and *z* directions, but is exponentially localised within a region of finite width, denoted *a*, in the *u* direction.

### Appendix A.1. Tensor Components

The non-zero Christoffel symbols for this metric are:

(A2) $$\begin{array}{cccc} \Gamma_{vu}^{v} & =\Gamma_{uv}^{v}=-\frac{1}{2u}-\frac{1}{2a}\frac{{\mathrm{sech}}^{2}\left(\frac{u}{a}\right)}{\tanh\left(\frac{u}{a}\right)} & \Gamma_{uu}^{u} & =-\frac{1}{2u}-\frac{1}{2a}\frac{{\mathrm{sech}}^{2}\left(\frac{u}{a}\right)}{\tanh\left(\frac{u}{a}\right)}\\ \Gamma_{vv}^{u} & =\frac{\gamma^{2}u}{2}\tanh^{2}\left(\frac{u}{a}\right)+\frac{\gamma^{2}u^{2}}{2a}{\mathrm{sech}}^{2}\left(\frac{u}{a}\right)\tanh\left(\frac{u}{a}\right) & \Gamma_{yy}^{u} & =\Gamma_{zz}^{u}=\frac{\gamma u}{2a}\frac{{\mathrm{sech}}^{2}\left(\frac{u}{a}\right)}{\tanh\left(\frac{u}{a}\right)}\\ \Gamma_{uy}^{y} & =\Gamma_{yu}^{y}=\Gamma_{uz}^{z}=\Gamma_{zu}^{z}=\frac{-1}{2a}\frac{{\mathrm{sech}}^{2}\left(\frac{u}{a}\right)}{\tanh\left(\frac{u}{a}\right)}. & \; \end{array}$$

Likewise, the non-zero Riemann tensor components are given by:

(A3) $$\begin{array}{cc} R_{vu}^{vu} & =-R_{uv}^{vu}=-R_{vu}^{uv}=R_{uv}^{uv}=-\frac{1}{a}{\mathrm{sech}}^{2}\left(\frac{u}{a}\right)+\frac{\left|u\right|}{a^{2}}\tanh\left(\frac{\left|u\right|}{a}\right){\mathrm{sech}}^{2}\left(\frac{u}{a}\right)\\ R_{vy}^{vy} & =-R_{yv}^{vy}=R_{yv}^{yv}=-R_{vy}^{yv}=\frac{1}{4a}{\mathrm{sech}}^{2}\left(\frac{\left|u\right|}{a}\right)-\frac{\left|u\right|}{4a^{2}}\tanh\left(\frac{\left|u\right|}{a}\right){\mathrm{sech}}^{2}\left(\frac{\left|u\right|}{a}\right)+\frac{\left|u\right|}{4a^{2}}\frac{{\mathrm{sech}}^{2}\left(\frac{\left|u\right|}{a}\right)}{\tanh\left(\frac{\left|u\right|}{a}\right)}\\ R_{uy}^{uy} & =-R_{yu}^{uy}=R_{yu}^{yu}=-R_{uy}^{yu}=\frac{1}{4a}{\mathrm{sech}}^{2}\left(\frac{\left|u\right|}{a}\right)-\frac{\left|u\right|}{2a^{2}}\tanh\left(\frac{\left|u\right|}{a}\right){\mathrm{sech}}^{2}\left(\frac{\left|u\right|}{a}\right)-\frac{\left|u\right|}{2a^{2}}\frac{{\mathrm{sech}}^{2}\left(\frac{\left|u\right|}{a}\right)}{\tanh\left(\frac{\left|u\right|}{a}\right)}\\ R_{yz}^{yz} & =-R_{zy}^{yz}=R_{zy}^{zy}=-R_{yz}^{zy}=+\frac{\left|u\right|}{4a^{2}}\tanh\left(\frac{\left|u\right|}{a}\right){\mathrm{sech}}^{2}\left(\frac{\left|u\right|}{a}\right)-\frac{\left|u\right|}{4a^{2}}\frac{{\mathrm{sech}}^{2}\left(\frac{\left|u\right|}{a}\right)}{\tanh\left(\frac{\left|u\right|}{a}\right)}. \end{array}$$

Finally, the non-zero Ricci tensor components are:

(A4) $$\begin{array}{cc} R_{v}^{v} & =\frac{\gamma }{2a^{2}}\left(-a{\mathrm{sech}}^{2}\left(\frac{u}{a}\right)+\frac{u{\mathrm{sech}}^{4}\left(\frac{u}{a}\right)}{\tanh\left(\frac{u}{a}\right)}+2u\tanh\left(\frac{u}{a}\right){\mathrm{sech}}^{2}\left(\frac{u}{a}\right)\right)\\ R_{u}^{u} & =-\frac{\gamma }{2a^{2}}{\mathrm{sech}}^{2}\left(\frac{u}{a}\right)\left(a+\frac{2u}{\tanh\left(\frac{u}{a}\right)}\right)\\ R_{y}^{y} & =R_{z}^{z}=-R_{u}^{u}. \end{array}$$

The Ricci scalar equals $R=R_{v}^{v}-R_{u}^{u}$. Note that despite the appearance of the absolute value |u|, all terms in Equations (A3) and (A4) are smooth and remain finite throughout spacetime.

### Appendix A.2. The Semi-Classical Limit

The constant γ in the definition of the metric can be related to the non-zero mass density $\rho_{0}$ of the planetary object by comparing the geodesic for a light point-particle μ in radial free fall, to the corresponding Newtonian equation of motion for a test-mass near the planet [9]. For massive time-like trajectories, we impose the Euler Lagrange equations to obtain the geodesics:

(A5) $$\begin{array}{c} \gamma |u|\dot{v}=C_{1}\;\dot{u}=\pm \sqrt{C_{1}^{2}-\gamma \left|u\right|}. \end{array}$$

Here, $\partial_{\sigma }C_{1}=0$. This equation is solvable and results in $\left|u\right|=-\frac{\gamma }{4}\sigma^{2}\pm c_{0}\sigma +u_{0}$. Here, we defined $u\left(\sigma =0\right)=u_{0}$ with $u_{0}$ a positive initial position and $|\dot{u}\left(\sigma =0\right)|=c_{0}=\sqrt{C_{1}^{2}-\gamma \left|u_{0}\right|}$ with $\pm c_{0}$ the initial velocity. The parameters $u_{0}$ and $c_{0}$ can be used to match the initial conditions for any given geodesic. Taking the second derivative yields $\frac{d^{2}u}{d\sigma^{2}}=-\mathrm{sgn}\left(u\right)\frac{\gamma }{2}$. This result can be compared to the Poisson equation, which is given in the semi-classical limit by $\mu \frac{d^{2}u}{dt^{2}}=\mathrm{sgn}\left(u\right)2\pi G\mu \rho_{0}$. We thus arrive at the relation $\gamma =4\pi G\rho_{0}$.

### Appendix A.3. Equations of State

In the static frame, we derive the position-dependent density ρ(u) and pressure P(u) by solving the Einstein equations $G_{\mu \nu }=8\pi GT_{\mu \nu }$ and subsequently, calculating the relevant components of $T_{\mu \nu }$.

In the static frame of the flat planet, $T_{v}^{v}$ equals the energy density ρ, while $T_{u}^{u}$, $T_{y}^{y}$, and $T_{z}^{z}$ are pressures $P_{i}$ with i∈u,y,z, and $P_{y}=P_{z}$ by symmetry. From the expressions for the tensor elements given above we can see that the density of the flat planet is given by the function:

$$\begin{array}{cc} \rho = & \frac{\gamma }{16\pi a^{2}}{\mathrm{sech}}^{2}\left(\frac{u}{a}\right)\left(-a+\frac{3}{2}u\tanh\left(\frac{u}{a}\right)+\frac{5}{2}u/\tanh\left(\frac{u}{a}\right)\right), \end{array}$$

Likewise, the pressure is given by:

$$\begin{array}{cc} P_{u} & =-P_{y}=-P_{z}=\frac{-\gamma }{16\pi a^{2}}{\mathrm{sech}}^{2}\left(\frac{u}{a}\right)\left(-a+\frac{1}{2}u\tanh\left(\frac{u}{a}\right)-\frac{1}{2}u/\tanh\left(\frac{u}{a}\right)\right). \end{array}$$

The negative ratio $P_{u}/P_{y}=-1$ corresponds to that of the equation of state for an electrical field in the *u* direction, with electromagnetic field strength tensor $F_{tu}=-F_{ut}=E_{u}$ [16]. We can thus consider a decomposition of the energy–momentum tensor for the planetary object of the form:

(A6) $$\begin{array}{cc} T_{\nu }^{\mu }= & {T_{\nu }^{\mu }}^{\left(M\right)}+{T_{\nu }^{\mu }}^{\left(E\right)}=\left(P^{M}+\rho^{M}\right)u^{\mu }u_{\nu }-P^{M}\delta_{\nu }^{\mu }+\left(F^{\mu \alpha }F_{\alpha \nu }-\frac{1}{4}\delta_{\nu }^{\mu }F_{\alpha \beta }F^{\alpha \beta }\right)\\ = & \mathrm{Diag}\left[\rho^{M}+\frac{E_{u}^{2}}{2},\frac{E_{u}^{2}}{2},-\frac{E_{u}^{2}}{2},-\frac{E_{u}^{2}}{2}\right]. \end{array}$$

Here, we assumed that the pressure $P^{M}$ due to the massive particles making up the planetary object is zero. This would be appropriate for motionless dust and negligible coupling between the mass and the electromagnetic field [16]. The equations of state derived from the ratios between the pressures and density are displayed in the left lower panel of Figure A1. In the upper panels, the metric and energy–momentum tensor components themselves are visualised, while the lower right panel displays the Ricci scalar.

We then find expressions for $\rho^{M}$ and $\rho^{E}=\frac{E_{u}^{2}}{2}$ given by:

(A7) $$\begin{array}{cc} \rho^{M} & =+\frac{\gamma }{8\pi a^{2}}{\mathrm{sech}}^{2}\left(\frac{u}{a}\right)\left(-a+u(\tanh\left(\frac{u}{a}\right)+1/\tanh\left(\frac{u}{a}\right)\right)\\ \rho^{E} & =-\frac{\gamma }{16\pi a^{2}}{\mathrm{sech}}^{2}\left(\frac{u}{a}\right)\left(-a+\frac{u}{2}(\tanh\left(\frac{u}{a}\right)-1/\tanh\left(\frac{u}{a}\right)\right). \end{array}$$

This solution to the Einstein equations thus corresponds to the density and pressure of a motionless dust with a background electromagnetic field in the *u*-direction. This ‘planet’ is non-singular, static, and it has non-zero mass. In this construction, the electromagnetic field comes from an antisymmetric charge distribution around u=0, with a negligible coupling between the mass and the electromagnetic field [16]. The radiation pressure of the electromagnetic field precisely keeps the mass from falling inward, making the solution static.

Note that we do not claim these densities constitute a realistic scenario for any actual gravitational object, just like the assumption of infinite uniform extent in the *y* and *z* directions was not realistic to begin with. The toy model instead serve the purpose of showing that the *a* metric leading to the black brane could in principle be constructed out of well-understood gravitational components, and does not violate any laws of nature, nor introduce any sort of new physics.

### Appendix A.4. Gravitational Collapse

The toy model for the planetary object is a static solution of the Einstein equations and admits a consistent physical interpretation in terms of massive dust and a specific electric field. The pressure from the electromagnetic field cancels the gravitational forces and keeps the planet from collapsing inwards. However, if the mass of the system is increased slightly, the electromagnetic field pressure will no longer suffice to cancel the gravitational pull. The resulting inwards motion of mass will not increase the electromagnetic field strength at u=0, as long as the total amount of charge in the system does not change. The instability thus worsens over time and the configuration is intrinsically unstable to small changes in mass or charge. That is, the board-like planetary object inevitably undergoes gravitational collapse.

Unfortunately, we cannot solve for the dynamics of the gravitational collapse exactly. It is possible, however, to approximately represent subsequent stages of the collapse process by a series of static toy model metrics, each using a different (decreasing) value of the planetary width *a*, but keeping the total energy the same for all metrics. To impose the constant energy requirement, we consider the conserved energy associated with the current through a space-like hypersurface [17,18]:

(A8) $$\begin{array}{cc} E_{R} & =\frac{1}{4\pi G}\int_{\Sigma }d^{3}\vec{u}\sqrt{\gamma }n_{\mu }J_{R}^{\mu }=\frac{1}{2\pi g}\int_{\delta \Sigma }d^{2}\vec{u}\sqrt{\gamma^{\left(2\right)}}n_{\mu }\sigma_{\nu }\nabla^{\mu }K^{\nu }. \end{array}$$

Here, we used Stokes’s theorem and $J_{R}^{\mu }=\nabla_{\nu }\left(\nabla^{\mu }K^{\nu }\right)$ with time-like Killing vector $K^{\nu }$. The hypersurface δΣ is taken at u→∞ and $\sigma^{\mu }$ is its outward-pointing normal vector. The hypersurface Σ itself has normal vector $n^{\mu }$. In the metric of Equation (A1), these vectors have the non-zero components:

(A9) $$\begin{array}{ccc} \; & n_{0}=-{\left(\gamma u\tanh(\frac{u}{a})\right)}^{1/2}\; & \sigma_{1}=+{\left(\gamma u\tanh(\frac{u}{a})\right)}^{-1/2}. \end{array}$$

Using also that $K^{\nu }=\left(1,0,0,0\right)$, results in:

(A10) $$\begin{array}{cc} n_{\mu }\sigma_{\nu }\nabla^{\mu }K^{\nu } & =-\nabla^{0}K^{1}=-g^{00}\Gamma_{00}^{1}K^{0}=\frac{\gamma }{2}\tanh\left(\frac{u}{a}\right)+\frac{\gamma u}{2a}{\mathrm{sech}}^{2}\left(\frac{u}{a}\right). \end{array}$$

The metric for the two-dimensional hypersurface δΣ is given by $\gamma_{ij}^{\left(2\right)}du^{i}du^{j}=\left(dy^{2}+dz^{2}\right)/\tanh\left(\left|u\right|/a\right)$. With this, the Komar integral becomes:

(A11) $$\begin{array}{c} E_{R}=\frac{1}{2\pi G}\int_{\delta \Sigma }dydz\left(\frac{\gamma }{2}+\frac{\gamma u}{2a}\frac{{\mathrm{sech}}^{2}\left(\frac{u}{a}\right)}{\tanh(\frac{\left|u\right|}{a})}\right)=\frac{\gamma }{4\pi G}\int dydz\equiv M. \end{array}$$

Here, we used the fact that neither of the two terms in the brackets depends on *y* or *z* to take them out of the integral, and we evaluated the remaining expression at u=∞. The resulting value of $E_{R}$ is positive, independent of *a*, and equal to the total mass of the planetary object. The total energy can thus be kept constant in a sequence of metrics with different values of *a* by keeping *M* constant.

Similarly, the total charge can be calculated using $Q=-2\int_{\delta \Sigma }dydz\sqrt{\gamma^{\left(2\right)}}n_{\mu }\sigma_{\nu }F^{\mu \nu }$. This results in a zero total charge, consistent with the anti-symmetric charge distribution identified before. The value of the electric field at u=0 required to maintain a static solution, however, is given by $E^{\rho }\left(u=0\right)=\sqrt{\frac{3\gamma }{8\pi a}}$ and increases uniformly with decreasing *a*. Therefore, if we take the static solution at any value of *a* and keep the charge *Q* fixed while increasing the mass slightly, the electric field strength at u=0 will necessarily be smaller than that required to cancel the gravitational pull of the increased mass. The obtained configuration, therefore, is not static and starts to collapse inwards. Since the electric field strength required to balance the gravitational pull increases as *a* decreases, the electric field cannot stop the inward motion of the massive dust, and the flat planet collapses into a singular, infinitely thin, massive brane at u=0.

To reach this final state as the result of any explicit dynamical process, we would need to consider some way for the massive particles to interact. After all, for completely non-interacting particles, the collapsing dust will simply move past u=0, come out on the other side, and perform oscillatory motion due to momentum conservation. In the presence of weak interactions providing dissipation of kinetic energy, however, the singular final configuration is unavoidable.

Note that the value of the mass density $\rho^{M}$ in the flat planet precisely vanishes at u=0 for any non-zero value of *a*. There thus seems to be no build-up of mass at u=0 as the planet collapses inwards. However, expanding the expression for $\rho^{M}$ for u=δu≪a to lowest order in δu/a yields $\rho^{M}\left(\delta u\right)\approx \frac{\gamma }{6\pi a^{3}}\delta u^{2}$. Taking both δu and *a* to zero together, we can keep their ratio fixed by requiring a=dδu with d≫1. The mass density then reduces to $\rho^{M}\left(\delta u\right)=\frac{\gamma }{6\pi }\frac{1}{d\delta u}$. This shows that there is a diverging amount of mass within the shell [−a,a] in the limit a→0, even if the mass density precisely at u=0 always vanishes. In other words, the collapsing board-like planetary object results in diverging amounts of mass accumulating arbitrarily close to u=0, creating a physical singularity, or black brane.

**The horizon**—The metric of Equation (A1) in the limit of a→0 describes the toy model black brane. Since the position of the singularity in this metric is fixed at u→0 for all times *v*, the u,v coordinates are suitable for describing static observers who maintain a fixed distance to the black-brane.

Starting from the geodesic Equation (A5), we can express the trajectory of a massive in-falling test particle as:

(A12) $$\begin{array}{cc} \; & \left|v\right|=-\mathrm{sgn}\left(u\right)\frac{2}{\gamma }\mathrm{arctanh}\left(\frac{\sqrt{C_{1}^{2}-\gamma \left|u\right|}}{C_{1}}\right)+v_{0}. \end{array}$$

As shown in the right panel of Figure A2, a static observer (using u,v coordinates) will see an in-falling test mass approach the black brane forever, without reaching the horizon at $u=0^{+}$ in any finite time *v*. Crucially, this implies that for all static observers at position $u_{0}>0$, the region u<0 is inaccessible. In other words, the horizon at $u=0^{+}$ separates spacetime into disconnected regions, even though the black brane singularity is naked.

Conversely, the in-falling test mass can also be described in a co-moving frame. In the limit a→0, the transformation from u,v coordinates to those used by an inertial (free-falling, $c_{0}=v_{0}=0$) observer are the same as those identified in the usual discussion of the Rindler and Minkowski metrics. Using those, we see that the proper frame for a free-falling observer is given by the Minkowski metric (for all u≠0), in which the observer appears static. We can also consider a mixed frame, in which the time coordinate *v* is transformed to the proper time *t* of the free-falling observer, but distance are still given in terms of the *u* coordinate. As shown in the left panel of Figure A2, it then becomes clear that the observer will reach the black hole horizon in finite proper time *t*.

**Surface gravity**—Using the metric of Equation (A1) in the limit a→0, the geodesics of (massless) light can be derived by demanding $L=\gamma |u|{\dot{v}}^{2}-\frac{1}{\gamma |u|}{\dot{u}}^{2}=0$. This yields the light trajectories in the u,v coordinates, given by:

(A13) $$\begin{array}{ccc} \; & \dot{v}=\pm \frac{1}{\gamma |u|}\dot{u}\; & \gamma \left(v-v_{0}\right)=\pm sgn\left(u\right)\ln\left(\gamma |u|\right). \end{array}$$

Here, the dot is defined to be the derivative with respect to the affine parameter σ. Using these expressions, the retarded ($t_{r}$) and advanced ($t_{a}$) times for the null-geodesics can be defined as:

(A14) $$\begin{array}{ccc} \; & t_{r}=vs.-\frac{sgn(u)}{\gamma }\ln\left(\gamma x\right)\; & t_{a}=vs.+\frac{sgn(u)}{\gamma }\ln\left(\gamma x\right) \end{array}$$

The metric can then be expressed in terms of the retarded time and position as $ds^{2}=-\gamma \left|u\right|dt_{r}^{2}-dudt_{r}-dt_{r}du$. From here, we can calculate the surface gravity of the black hole, which is defined by $K_{;b}^{a}K^{b}=-\kappa K^{a}$, where κ is the surface gravity on the horizon and $K^{a}$ is the time-like Killing vector associated with the retarded time [17,19]. To solve the Killing equation, we use the non-zero Christoffel symbols for the metric in the limit a→0:

(A15) $$\begin{array}{ccccc} \; & \Gamma_{uu}^{u}=\frac{-\gamma }{2}\frac{x}{\left|x\right|}\; & \Gamma_{xu}^{u}=\Gamma_{ux}^{u}=\frac{1}{2}\frac{x}{{\left|x\right|}^{2}}\; & \; & \Gamma_{xu}^{x}=\Gamma_{ux}^{x}=\frac{\gamma }{2}\frac{x}{\left|x\right|}. \end{array}$$

This results in the relation $\kappa =\frac{\gamma }{2}$, showing that the black brane has finite, non-zero, and constant surface gravity.
